# Identification of *Listeria monocytogenes* Determinants Required for Biofilm Formation

**DOI:** 10.1371/journal.pone.0113696

**Published:** 2014-12-17

**Authors:** Almaris N. Alonso, Kyle J. Perry, James M. Regeimbal, Patrick M. Regan, Darren E. Higgins

**Affiliations:** 1 Harvard Medical School, Department of Microbiology and Immunobiology, 77 Avenue Louis Pasteur, Boston, Massachusetts, 02115, United States of America; 2 Food and Drug Administration–Winchester Engineering and Analytical Center, 109 Holton St., Winchester, Massachusetts, 01890, United States of America; University of Illinois at Chicago College of Medicine, United States of America

## Abstract

*Listeria monocytogenes* is a Gram-positive, food-borne pathogen of humans and animals. *L. monocytogenes* is considered to be a potential public health risk by the U.S. Food and Drug Administration (FDA), as this bacterium can easily contaminate ready-to-eat (RTE) foods and cause an invasive, life-threatening disease (listeriosis). Bacteria can adhere and grow on multiple surfaces and persist within biofilms in food processing plants, providing resistance to sanitizers and other antimicrobial agents. While whole genome sequencing has led to the identification of biofilm synthesis gene clusters in many bacterial species, bioinformatics has not identified the biofilm synthesis genes within the *L. monocytogenes* genome. To identify genes necessary for *L. monocytogenes* biofilm formation, we performed a transposon mutagenesis library screen using a recently constructed *Himar1 mariner* transposon. Approximately 10,000 transposon mutants within *L. monocytogenes* strain 10403S were screened for biofilm formation in 96-well polyvinyl chloride (PVC) microtiter plates with 70 *Himar1* insertion mutants identified that produced significantly less biofilms. DNA sequencing of the transposon insertion sites within the isolated mutants revealed transposon insertions within 38 distinct genetic loci. The identification of mutants bearing insertions within several flagellar motility genes previously known to be required for the initial stages of biofilm formation validated the ability of the mutagenesis screen to identify *L. monocytogenes* biofilm-defective mutants. Two newly identified genetic loci, *dltABCD* and *phoPR*, were selected for deletion analysis and both Δ*dltABCD* and Δ*phoPR* bacterial strains displayed biofilm formation defects in the PVC microtiter plate assay, confirming these loci contribute to biofilm formation by *L. monocytogenes*.

## Introduction

*Listeria monocytogenes* is a Gram-positive, food-borne pathogen that causes gastroenteritis in healthy individuals that can develop into a severe invasive illness in the elderly, pregnant women, infants, and the immunocompromised [1]. *L. monocytogenes* is a significant threat for contamination of ready-to-eat (RTE) foods, as bacteria can persist within food-processing plants and grow at refrigeration temperatures. *L. monocytogenes* has been the cause of the most severe food-borne disease outbreaks in the U.S. Most recently, in 2011 *L. monocytogenes* contaminated cantaloupes were responsible for sickening 147 individuals and resulted in 33 deaths [2]. *L. monocytogenes* can adhere to multiple biotic and abiotic surfaces and persist within biofilms [3] to facilitate contamination of food supplies. Furthermore, the ability of bacteria to replicate at low temperatures (<4°C) and survive for long periods within the environment under adverse conditions has made *L. monocytogenes* a major concern for the manufacturing and food processing industries [4]. Nonetheless, despite these concerns relatively little is known about the genetic determinants for biofilm formation by *L. monocytogenes*.

Biofilms are structured communities of bacterial cells adherent to an inert or living surface [5]. Biofilm-coated surfaces are a challenge to decontaminate as bacteria in biofilms are more resistant to detergents and biocides than planktonic bacteria [3], [6]. Extracellular polymeric substances (EPS), a hallmark of biofilm formation, participate in the formation of the microbial aggregates that make up a biofilm [7]-[9]. *L. monocytogenes* biofilms firmly attach bacteria to glass, plastic, and steel [3], [10], [11]. *L. monocytogenes* adhere more strongly to polymers than other biofilm-forming food-borne pathogens and the efficiency of *L. monocytogenes* attachment has been shown to be dependent on the properties of the substratum [12].

Transposon mutagenesis remains one of the most useful tools in bacterial genetic analyses, facilitating the discovery and investigation of gene function and regulation [13], [14]. Although several *L. monocytogenes* genes have been previously identified as being required for biofilm formation using transposon mutagenesis approaches [15], [16], the transposon delivery vectors used did not allow for optimal transposon library complexity and possessed the potential for multiple transposon insertions per mutant. A recently published *Himar1* transposon system for *L. monocytogenes* allows greater transposon library complexity due to genome-wide insertion coverage with no discernable transposon insertional hotspot bias and a single transposition event per generated mutant [17].

In this report, we describe to date the most comprehensive transposon mutagenesis screen for *L. monocytogenes* biofilm deficient mutants. A total of 38 genetic loci were identified to be involved in *L. monocytogenes* biofilm formation. Two of these loci, the D-alanylation pathway genes *dltABCD* and the phosphate-sensing two-component system *phoPR* were investigated further for their importance in biofilm formation. We constructed *L. monocytogenes phoPR* and *dltABCD* deletion strains to confirm the requirement of these genetic loci for biofilm formation. Our results indicated a statistically significant reduction in biofilm formation by the *ΔdltABCD* and *ΔphoPR* strains compared to wild-type bacteria in the PVC microtiter plate assay and by confocal scanning laser microscopy.

## Materials and Methods

### Bacterial strains

Bacterial strains and plasmids used in this study are listed in Table S1 in S1 File. Primers used in this study are listed in Table S2 in S1 File. *Escherichia coli* strains were grown in Luria-Bertani medium. *Listeria monocytogenes* strains were grown in brain-heart infusion (BHI; Difco, Detroit, MI) medium, tryptic soy broth yeast extract (TSBYE; 3.0% tryptic soy broth (BD, Franklin Lakes, NJ) and 0.6% yeast extract (BD)) medium, and Hsiang-Ning Tsai medium (HTM) [18]. All bacterial stocks were stored at -80°C in BHI supplemented with 40% glycerol. The following antibiotics were used at the indicated concentrations: carbenicillin, 100 µg/mL; streptomycin, 100 µg/mL; erythromycin, 3 µg/mL; chloramphenicol, 7.5 µg/mL (*L. monocytogenes*) or 20 µg/mL (*E. coli*) (Sigma-Aldrich, St. Louis, MO).

### Arraying the *Himar1* library and biofilm formation screen

An aliquot of DP-L5539 was grown at 37°C with shaking (200 rpm) in BHI with erythromycin for 24 hours and plated onto BHI + erythromycin plates to allow isolation of single colonies. Approximately 10,000 individual colonies were picked and arrayed into deep-well 96-well plates containing BHI + erythromycin and grown for 16 hours at 25°C without shaking. Aliquots of bacterial cultures were then mixed with sterile glycerol to a final concentration of 40% glycerol and transferred to 96-well plates for storage at −80°C.

### Quantitative assay for biofilm formation

Biofilm formation assays were performed as previously described with minor modifications [3]. Briefly, aliquots of the arrayed DP-L5539 library were inoculated into TSBYE medium (BD, Franklin Lakes, NJ) in 96-well plates (BD, Franklin Lakes, NJ) and grown statically at 35°C for 24 hours. The arrayed cultures were then diluted 1:10 into freshly made HTM medium [18] with 3% glucose and 0.1 mg/mL each cysteine and methionine in new 96-well PVC microtiter plates. Plates with lids were wrapped with parafilm to minimize evaporation and incubated statically for 96 hours at 35°C. Following growth, planktonic cells and loosely adhered bacteria were removed by manual pipetting up and down four times. The plates were then washed three times with sterile double-distilled water using a Cellwasher 600 instrument (Skatron, Sterling, VA). Plates were allowed to air-dry for 1 hour at 42°C and then stained with 0.1% crystal violet solution (Harelco, Gebbstown, NJ) for 45 min. Plates were washed with sterile double-distilled water as previously described and allowed to air-dry for 45 min at 42°C. To quantify biofilm formation, deposited crystal violet was solubilized by adding 150 µL of 33% acetic acid (Amresco, Solon, OH) for 15 min, pipetting up and down several times, and the OD_595_ was measured using a SPECTRAmax M2 plate reader equipped with SOFTmax Pro software (Molecular Devices). Transposon mutants for which the OD_595_ was at least two standard deviations less than the plate average were rescreened as above in octuplicate and the OD_595_ of each mutant averaged. Transposon mutants for which the OD_595_ was at least two standard deviations less than wild-type bacteria were identified as biofilm formation mutants.

### Plasmid and strain construction

In-frame *dltABCD* and *phoPR* deletion constructs were produced by SOE PCR as previously described [19]. The resulting Δ*dltABCD* and Δ*phoPR* PCR products were ligated into pKSV7 using the *SalI/BamHI* and *BamHI/EcoRI* restriction sites, respectively, to generate pKSV7 Δ*dltABCD* and pKSV7 Δ*phoPR*, respectively. pKSV7 Δ*dltABCD* and pKSV7 Δ*phoPR* were electroporated into 10403S and allelic exchange was performed as previously described [20] to generate strains DH-L2054 and DH-L2055, respectively. All PCR amplifications for cloning were performed using *PfuTurbo* DNA Polymerase AD (Agilent Technologies, Inc, Santa Clara, CA) as per the manufacturer's instructions. All plasmids and strains were verified by DNA sequencing.

### Flagellar motility assay

Putative biofilm formation mutants were grown for 96 hours in HTM as previously described and then inoculated by sterile toothpick into 100 mm petri dishes containing Bacto motility agar (BD, Franklin Lakes, NJ) and incubated at 30°C. The diameter of bacterial halos in the agar was then measured following 48 hours of growth.

### Identification of transposon insertion sites

*Himar1* insertion sites were identified by amplifying the insertion junctions using a two-round semi-arbitrary PCR as previously described [21] with minor modifications. Briefly, bacteria from single colonies of biofilm formation mutants grown on BHI agar plates containing erythromycin were used as templates in 25 µL PCR reactions using RedTaq Readymix PCR Reaction Mix (Sigma-Aldrich, St. Louis, MO) as per the manufacturer's instructions with primers ARB1 and marK3 (Table S2 in S1 File) using PCR program #1. One microliter of the first-round PCR was then used as template for a second-round PCR reaction as indicated above with primers ARB2 and marK4 (Table S2 in S1 File) using PCR program #2. PCR products from the second PCR reaction were purified using the QIAquick PCR Purification Kit (Qiagen, Valencia, CA) as per the manufacturer's instructions and submitted to the Dana-Farber/Harvard Cancer Center DNA Resource Core (http://dnaseq.med.harvard.edu) at Harvard Medical School (Boston, MA) for sequencing with primer marK4. PCR program #1: 1 cycle: 91°C for 2 min; 6 cycles: 91°C for 15 sec, 29°C for 15 sec, 72°C for 75 sec; 30 cycles: 91°C for 15 sec, 52°C for 15 sec, 72°C for 75 sec; 1 cycle: 72°C for 5 min. PCR program #2: 1 cycle: 91°C for 2 min; 35 cycles: 91°C for 15 sec, 52°C for 15 sec, 72°C for 2 min; 1 cycle: 72°C for 5 min.

### Transmission electron microscopy

TEM was performed using ruthenium red to stain the extracellular polymeric matrix produced by *L. monocytogenes*. Bacterial samples were fixed in a 0.1 M sodium cacodylate buffer (pH 7.4) containing 2.5% glutaraldehyde, 1.25% paraformaldehyde, and 0.03% picric acid for 2 hours at 25°C. 0.5% ruthenium red (RR) (Electron Microscopy Sciences, Hatfield, PA) was added to the fixed samples and cells incubated for 3 hours at 25°C. Cells were centrifuged for 3 min at 3,000 rpm, bacterial pellets were washed 3 times with 0.1 M cacodylate buffer and 0.5% RR and incubated with 1% OsO_4_ + 0.5% RR for 2 hours. Samples were then washed in double-distilled water 3 times and incubated in 1% aqueous uranyl acetate for 1 hour followed by dehydration in increasing concentrations of ethanol (10 min each; 50%, 70%, 90%, 2 × 10 min in 100%). The samples were then placed in propylene oxide for 1 hour, followed by 16 hour incubation in a 1:1 mixture of propylene oxide and Spurr's Low Viscosity Embedding media (Electron Microscopy Sciences, Hatfield, PA). The following day the samples were embedded in Spurr's Low Viscosity Embedding media and polymerized at 60°C for 48 hours. Ultrathin sections of 60 nm were cut on a Reichert Ultracut-S microtome. Sections were placed onto copper grids, stained with 0.2% lead citrate and examined in a JEOL 1200EX or a Tecnai G^2^ Spirit BioTWIN Transmission electron microscope and images were recorded with an AMT 2k CCD camera.

### Scanning electron microscopy

Commercially available bean sprouts (Jonathan's Organics) were used for scanning electron microscopy (SEM). Bean sprouts were autoclaved in sterile distilled water and placed in HTM minimum agar media with 3% glucose and inoculated with 10 µl of a 1:10 dilution of a *L. monocytogenes* 10403S culture grown statically for 24 hours at 37°C. Following 24 hours incubation at 37°C, sprout samples were rinsed twice with 0.1 M cacodylate buffer and then with 25% glutaraldehyde. Bean sprouts were then dehydrated with increasing concentrations of ethanol (30 min each; 30%, 50% and 70% ethanol), 85% ethanol for 24 hours, 95% ethanol for 48–72 hours and 100% ethanol for 72–96 hours. Samples were critical point dried and sputter coated with 1:4 Pt/Pd for 2 min and visualized using a Zeiss EVO 55 (Carl Zeiss AG - EVO 50 Series) scanning electron microscope.

### Confocal scanning laser microscopy

The specified strains were grown for 24 hours in TSBYE medium and the OD_600_ was determined to produce inoculating aliquots. An aliquot of each culture was diluted in HTM minimal media to obtain an OD_600_ of 0.05–0.06. Four milliliters of the HTM cultures were individually transferred to a 35 mm glass bottom culture dish with a 10 mm microwell cover glass (Mat Tek Corporation, Ashland, MA). Samples were incubated at 35°C for 96 hours. After incubation, 3 mL of culture media were removed from each glass bottom culture dish. Samples were rinsed gently with sterile distilled water to remove planktonic bacterial cells and stained using the LIVE/DEAD BacLight Bacterial Viability kit (L7007, Molecular Probes, Invitrogen). Briefly, a solution composed of 1.5 µL Component A mixed with 1.5 µL Component B in 997 µL of sterile distilled water was prepared. Three hundred microliters of the solution was added to the center of each glass bottom dish and samples were incubated for 15 min at 25°C in the dark. Each glass bottom dish was then imaged using a Nikon Ti w/A1R inverted confocal microscope using fluorescein and Texas red band-pass filters to visualize SYTO 9 and propidium iodide, respectively. Images were captured using NIS-Elements software (Nikon Instruments Inc., Melville, N.Y.) from six areas of each of the triplicate biofilm samples.

## Results

### *L. monocytogenes* strain 10403S firmly adheres to nutritive surfaces and produces EPS

**Figure 1:**
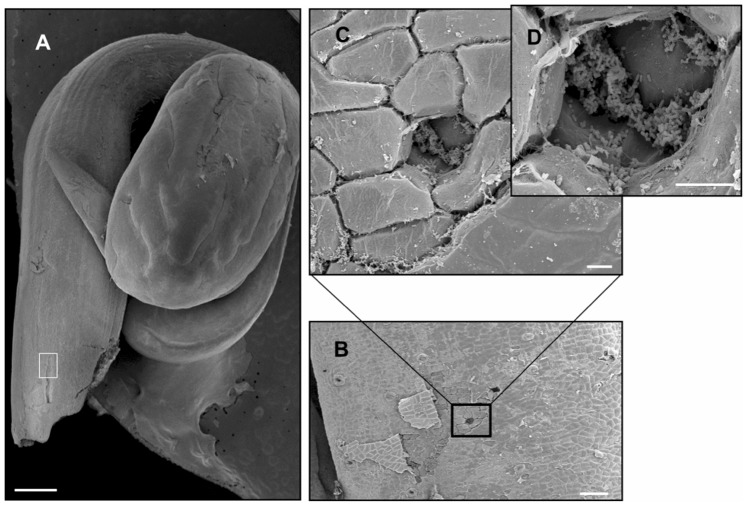
Scanning electron microscopy of a bean sprout inoculated with *L. monocytogenes*. Sterile bean sprouts were placed in HTM agar media with 3% glucose and inoculated with 10 µl of a 1:10 dilution of a 24-hour culture of 10403S. Following a 24 hour incubation, bean sprouts were processed for scanning electron microscopy (A) Bean sprout (bar = 1 mm) (B) magnified view of the white square from (A) (bar = 100 µm). (C) Bean sprout vegetative tissue colonized with *L. monocytogenes* (bar = 10 µm) (D) magnification of (C) (bar = 10 µm).

**Figure 2:**
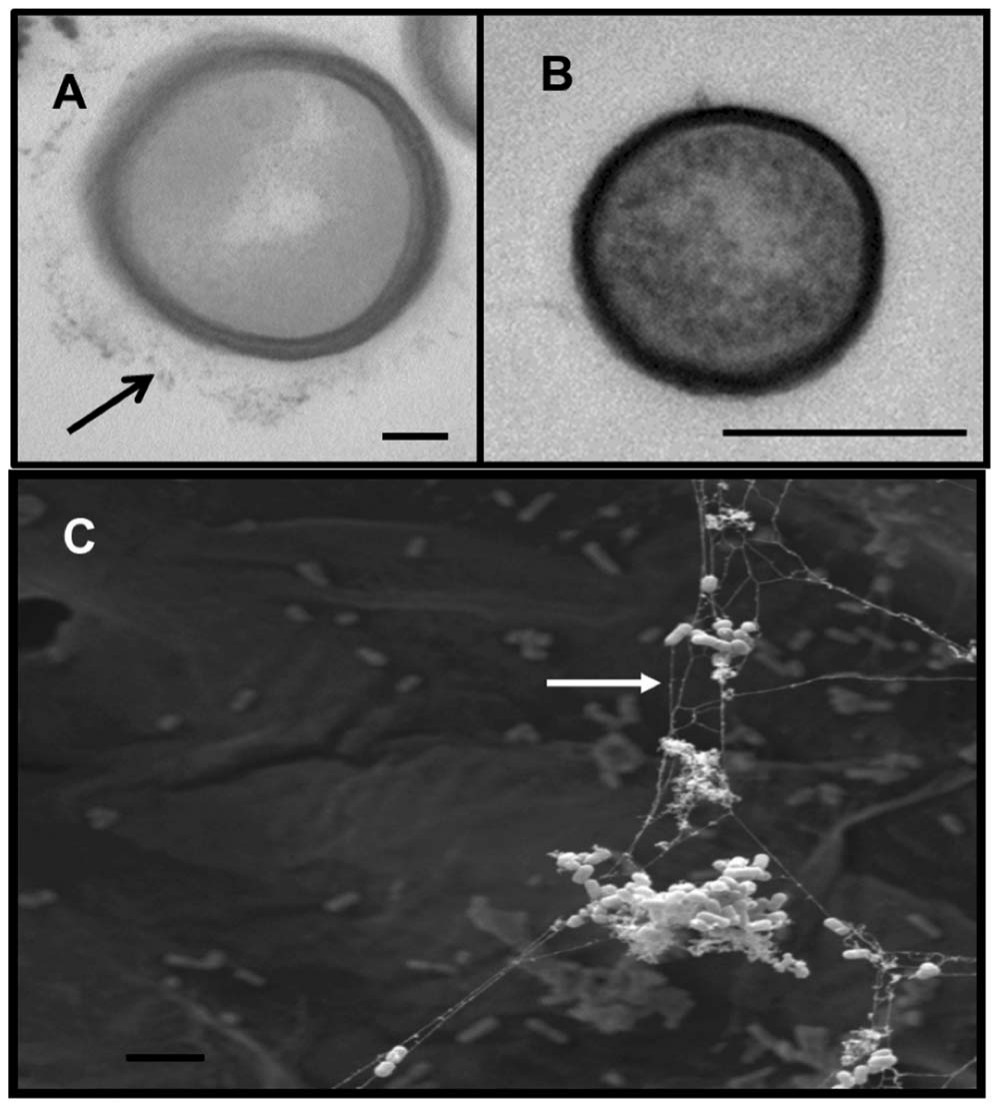
Transmission and scanning electron microscopy analysis of *L. monocytogenes* EPS production. *L. monocytogenes* 10403S bacteria in biofilms formed on dialysis tubing membranes (regenerated cellulose) (A) (bar = 100 nm) or planktonic bacteria grown in broth culture (B) (bar = 500 nm) were examined by TEM at 72 hours post-inoculation. (C) SEM of a *L. monocytogenes* biofilm developed on regenerated cellulose at 24 hours post-inoculation (bar = 10 µm). Arrows indicate EPS. For TEM, samples were fixed with 25% glutaraldehyde, rinsed with cacodylate buffer and stained with ruthenium red to visualize EPS material. For SEM, samples were rinsed with multiple dilutions of ethanol prior to visualization.

*L. monocytogenes* strain 10403S [22] has previously been used to study biofilm formation during growth of bacteria on PVC [3]. To validate the use of 10403S in the elucidation of *L. monocytogenes* biofilm determinants, we assessed the ability of 10403S bacteria to grow on two physiologically relevant surfaces, raw bean sprouts and regenerated cellulose. Following 24 hours of growth on sterilized bean sprouts, scanning electron microscopy (SEM) revealed that 10403S firmly adheres to vegetative material (bean sprouts) and forms biofilms (Fig. 1). Aggregates of bacteria were readily observed on the surface of bean sprouts (Fig. 1B–1D), demonstrating the ability of *L. monocytogenes* to adhere to and form biofilms on a RTE food and validating the use of *L. monocytogenes* strain 10403S to study biofilm formation. High magnification of SEM images revealed that bacterial aggregates were composed of multiple bacteria connected by strands of EPS (Fig. 1D). To further visualize EPS production by *L. monocytogenes* during bacterial growth in biofilms, transmission electron microscopy (TEM) of biofilms formed on regenerated cellulose and planktonic bacteria grown in broth culture was performed. TEM revealed that *L. monocytogenes* cells growing in a biofilm produced detectable EPS, while bacteria growing planktonically did not produce visible EPS (Fig. 2).

### A *Himar1* transposon mutagenesis screen for biofilm-deficient *L. monocytogenes* mutants

To identify *L. monocytogenes* genes involved in biofilm formation, we performed a *Himar1* transposon mutagenesis screen. Approximately 10,000 individual mutants from an aliquot of the DP-L5539 10403S *Himar1* transposon library [17] were arrayed, grown under biofilm-inducing conditions, and screened for biofilm formation using a 96-well microtiter plate assay [3]. A total of 70 *Himar1* insertion mutants were identified that yielded at least two standard deviations less biofilm production compared to the mean of the individual 96-well plate in two independent experiments. Semi-arbitrary PCR and DNA sequencing of *Himar1* insertion sites from the isolated mutants revealed 49 independent transposon mutants representing 38 distinct genetic loci (Table 1).

**Table 1:** Identified *L. monocytogenes* biofilm-formation genes.

Function Group	Genes^a^	*Himar1* insertion site^b^	Biofilm reduction relative to WT (%) ave±SD^c^	Number of insertions	Number of independent insertions
**Biosynthesis**					
	Glycosyltransferase	*lmrg_01693*	93±0.41	3	2
	Membrane sulfatase	*lmrg_00331*	94±0.36	10	4
	Asparagine synthase	*lmrg_01304*	92±0.19	2	1
	*dltA*	*lmrg_02073*	97±0.10	1	1
	*dltB*	*lmrg_02072*	94±0.21	1	1
	UDP-glucose 4-epimerase	*lmrg_01771*	91±0.43	9	4
	*purD*	*lmrg_02507*	87±0.14	1	1
	*purE*	*lmrg_02497*	90±0.28	1	1
	*purF*	*lmrg_02503*	90±0.11	2	2
	*purH*	*lmrg_02506*	90±0.28	2	1
	*purL*	*lmrg_02502*	100±0.21	1	1
	*purN*	*lmrg_02505*	100±0.57	1	1
	Isocitrate dehydrogenase	*lmrg_01401*	100±0.38	1	1
	2-oxovalerate dehydrogenase component E1	*lmrg_00823*	98±0.35	3	2
	Xanthine ribosyltransferase	*lmrg_01032*	90±0.21	1	1
	Aconitate hydratase	*lmrg_01325*	92±0.12	1	1
	Homoserine dehydrogenase	*lmrg_01700*	84±0.35	1	1
	Peptidoglycan N-acetylglucosamine deacetylase	*lmrg_00107*	99±0.06	1	1
	Adenylosuccinate synthetase	*lmrg_02457*	89±0.07	1	1
**Gene Regulation**					
	Signal peptidase I	*lmrg_00721*	100±0.34	2	1
	*phoR*	*lmrg_01748*	99±0.02	1	1
	GntR family regulator	*lmrg_01251*	90±0.21	1	1
	Putative rRNA methylase	*lmrg_01305*	87±0.33	1	1
	DNA polymerase	*lmrg_01402*	100±0.36	2	1
	Putative Rrf2 family regulator	*lmrg_01481*	70±0.28	1	1
	ATP synthase beta subunit F1	*lmrg_01719*	92±0.49	1	1
**General growth-defective**					
	*plsX*	*lmrg_00956*	84±0.21	2	1
	Catalase	*lmrg_01912*	97±0.47	5	3
**Unknown functions**					
	Hypothetical	*lmrg_00049*	85±0.42	1	1
	Hypothetical	*lmrg_02457*	89±0.03	1	1
	Adenyl synthase	*lmrg_02487*	100±0.35	1	1
	Efflux protein	*lmrg_01872*	95±0.12	1	1
	Hypothetical protein	*lmrg_01206*	87±0.14	1	1
**Motility**					
	*fliQ*	*lmrg_00365*	100±0.20	2	1
	*flaA*	*lmrg_00387*	88±0.21	1	1
	*fliI*	*lmrg_00405*	100±0.07	1	1
	Flagellar hook associated protein #2	*lmrg_00396*	95±0.14	1	1
	*motA*	*lmrg_01748*	98±0.14	1	1

a *Putative functions were obtained from http://www.broadinstitute.org/annotation/genome/listeria_group/MultiHome.html*.

b *Based on DNA homologies with the L. monocytogenes 10403S genome database; lmrg refers to genetic loci within strain 10403S*.

c *% Compared to wild-type L. monocytogenes 10403S biofilm formation in two independent experiments*.

*Himar1* insertions were recovered in five separate structural components of *L. monocytogenes* flagella: *flaA*, *fliI*, *fliQ*, *motA*, and *lrmg_00396*. As flagellar motility is required for initial surface attachment [3], the recovery of these transposon mutants validates the ability of the transposon screen to identify biofilm formation mutants. Additionally, several of the genetic loci and pathways identified by our improved screen were also identified by other groups as being important for *L. monocytogenes* biofilm formation: *lmrg_01693* glycosyltransferase [15], [16], *lmrg_00331* membrane sulfatase, *lmrg_01719* ATP synthase ß subunit F1 [15], purine biosynthesis genes *purD*, *purE*, *purF*, *purH*, *purL*, and *purN* [15]; D-alanylation genes *lmrg_02073* (*dltA*) and *lmrg_02072* (*dltB*) [15]; and *lmrg_01251* GntR family response regulator [23]. Excluding transposon insertions into two genetic loci which resulted in severe general growth defects (*lmrg_00956* (*plsX*) and *lmrg_01912* catalase), novel recovered mutants harbored transposon insertions in genes corresponding to two broad functional categories, biosynthesis and gene regulation (Table 1).

Of particular interest are the *dltABCD* and *phoPR* operons as these two genetic loci have been previously implicated in biofilm formation in other bacterial species. The *dlt* operon was identified by transposon insertions within *lmrg_02073* (*dltA*) and *lmrg_02072* (*dltB*) (Table 1). The *dlt* operon of Gram-positive bacteria comprises four genes (*dltA, dltB, dltC*, and *dltD*), which catalyze the incorporation of D-alanine residues into lipoteichoic acids [24]. Loss of D-alanylation of lipoteichoic acids alters bacterial cell surface charge and results in increased sensitivity to cationic antimicrobial peptides and reduced biotic attachment and biofilm production by *Staphylococcus aureus* and *L. monocytogenes* [25]–[27]. The recovery of *Himar1* insertions in *dltA* and *dltB* suggests that *L. monocytogenes* requires D-alanylation of extracellular lipoteichoic acids to maintain proper surface charge and allow attachment to abiotic surfaces. The phosphate-sensing two-component system encoded by the *phoPR* operon was identified by a transposon insertion in *lmrg_01748* (*phoR*). As phosphate-sensing two-component systems have been previously shown to regulate biofilm formation in multiple Gram-positive and Gram-negative bacterial species [28]–[31], we hypothesize that *L. monocytogenes* also uses changes in inorganic phosphate levels as an environmental signal to regulate biofilm production.

### *L. monocytogenes* Δ*phoPR* and Δ*dltABCD* strains are defective for biofilm formation

**Figure 3:**
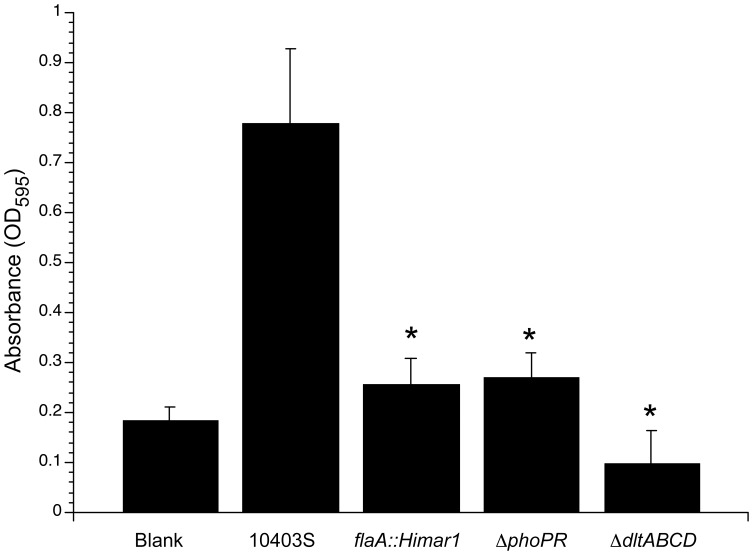
Biofilm formation by Δ*phoPR* and Δ*dltABCD L. monocytogenes*. Bacterial strains were inoculated into TSBYE media in 96-well plates and grown at 35°C for 24 hours. Cultures were then diluted 1:10 into fresh HTM media with 3% glucose and 0.1 mg/mL each cysteine and methionine in new 96-well PVC microtiter plates. Plates were incubated at 35°C for 96 hours, rinsed with dH_2_O using a semi-automated cell washer, stained with crystal violet, rinsed with acetic acid and the OD_595_ ±SD determined using a spectrophotometer. The data presented are representative of three independent experiments. *, p <0.05 (One-way ANOVA test).

To validate the requirement of *phoPR* and *dltABCD* for biofilm formation, we generated in-frame *phoPR* and *dltABCD* deletion strains and measured biofilm formation in PVC microtiter plates following 96 hours of growth under biofilm-inducing conditions (Fig. 3). Both the Δ*phoPR* and Δ*dltABCD* strains produced less biofilms, similar to the *flaA::Himar1* negative control strain, suggesting that the PhoPR two-component system and *dltABCD* gene products are necessary for biofilm formation by *L. monocytogenes*. To additionally assess biofilm formation and architecture, biofilms produced by Δ*phoPR* and Δ*dltABCD* bacteria were examined by confocal scanning laser microscopy (CSLM) (Table 2). Whereas *L. monocytogenes* 10403S produced a biofilm thickness of 31 ±1.69 µm by CSLM, both Δ*dltABCD* bacteria (17.00 ±1.84 µm) and Δ*phoPR* bacteria (15.28 ±0.65 µm) produced statistically thinner biofilms (p≤0.05, Student's *t-test*). This result indicates that both Δ*phoPR* and Δ*dltABCD* bacteria produced significantly less biofilms than the parental strain and therefore the PhoPR two-component system and *dltABCD* gene products are necessary for proper biofilm formation by *L. monocytogenes* 10403S.

**Table 2:** CSLM analysis of *L. monocytogenes* biofilm production.

Strain	Biofilm thickness (µm)^a^
L. monocytogenes 10403S	31.00 ± 1.69^b^
flaA::Himar1	22.00 ± 3.02^b^
ΔdltABCD	17.00 ± 1.84^b^
ΔphoPR	15.28 ± 0.65^b^

a Results presented are the means ±SD from two independent experiments performed in triplicate.

b Student's *t-test* indicated a statistically significant difference between biofilm thickness formed by *L. monocytogenes* 10403S compared to mutant bacterial strains (p ≤ 0.05).

## Discussion

In this study, *L. monocytogenes* 10403S was shown to firmly adhere to both nutritive and non-nutritive surfaces, bean sprouts and regenerated cellulose, respectively; and produce EPS, the hallmark of biofilm formation [5], [7] (Fig. 1 and Fig. 2). It is well established that not all microorganisms can adhere to non-nutritive surfaces such as PVC [32]. Accordingly, prior studies have shown that two Gram-positive cellulolytic soil bacteria, *Cellulomonas uda*, a facultative aerobe, and *Clostridium phytofermentans*, an obligate anaerobe, specifically adhered to nutritive surfaces as biofilms, but were unable to colonize non-nutritive surfaces [7], [32]. The capacity to adhere to multiple surfaces in the environment confers an ecological advantage to *L. monocytogenes* for occupying diverse niches, securing nutrients, and persisting in adverse conditions [7]. It has been shown that in regulated environments, such as food processing plants with established cleaning and sanitizing practices, bacterial biofilm formation on food contact surfaces is controlled [6]. However, microbial attachment can occur on non-food contact surfaces within these regulated environments. If left undisturbed, these attached microbes will form biofilms, generating a potential source for contamination of food with undesirable spoilage-causing or pathogenic bacteria [6].

In this study, we also report the most comprehensive transposon mutagenesis screen for *L. monocytogenes* biofilm formation genes to date. Approximately 10,000 independent *Himar1* insertion mutants were screened and 70 transposon insertion mutants deficient for biofilm formation comprising 38 distinct genetic loci identified (Table 1). The identification of five flagellar motility genes, which are known to be important for initial surface attachment during biofilm formation, and additional *L. monocytogenes* genes previously identified as important for biofilm formation validated the ability of our improved transposon mutagenesis screen to identify genes necessary for production of *L. monocytogenes* biofilms. The overlap between genetic loci identified in our and three separate *L. monocytogenes* biofilm production gene studies using diverse background strains further validates the findings of our current screen and may suggest near saturation of transposon screening to identify *L. monocytogenes* biofilm production genes. In addition, we further assessed the requirement of two identified genetic loci, *dltABCD* and *phoPR* for biofilm formation. Both Δ*phoPR* and Δ*dltABCD* bacteria produced significantly less biofilms in a microtiter plate assay (Fig. 3) and by confocal scanning laser microscopy analysis (Table 2). These data suggest that the D-alanylation of lipoteichoic acids mediated by the products of the *dltABCD* operon and the phosphate-sensing PhoPR two-component system play critical roles for biofilm formation by *L. monocytogenes*. Additional work is required to elucidate the specific role of D-alanylation of lipoteichoic acids and to determine how genes within the PhoPR regulon are necessary for biofilm formation.

Microbial attachment to surfaces has been attributed to both the nature of the polymer comprising the surface material and the cell surface characteristics of the bacterium [8], [9], [25]. A previous report suggested that the attachment of *S. aureus* to abiotic surfaces depends on the charge of the bacterial teichoic acids [33]. This study determined that a *dltA* mutant of *S. aureus* that lacked D-alanine within surface teichoic acids yielded bacteria with a higher negative charge and resulted in a biofilm-negative phenotype. The *S. aureus dltA* mutant exhibited a decrease in initial attachment to polystyrene or glass that was hydrophobic or negatively charged, respectively [33]. In *L. monocytogenes*, the *dltABCD* operon is also involved in the incorporation of D-alanine residues into lipoteichoic acids, resulting in a reduced negative charge on surface teichoic acids [26]. Additionally, loss of D-alanylation of teichoic acid was also found to decrease teichoic acid thickness and change envelope rigidity in Group B *Streptococcus* [34]. In this study, deletion of the *dltABCD* operon in *L. monocytogenes* resulted in a biofilm-deficient phenotype (Fig. 3). It is possible that reducing the amount of extracellular amino acids, such as D-alanine, would change the surface charge of *L. monocytogenes* [33] or alternatively change teichoic acid thickness or cell wall rigidity [34], thus decreasing the ability of bacteria to attach to the hydrophobic PVC surface used in this study.

The most common source of phosphorus in the environment is inorganic phosphate (P_i_). Phosphorus is an essential nutrient for all cells and is required for the biosynthesis of nucleotides, DNA, and RNA and for the functional regulation of protein activity by phosphorylation. Under phosphate starvation conditions, many bacteria induce the synthesis of proteins that facilitate efficient use of limited phosphate resources and make alternative sources of phosphorous accessible [28]. In the closely related Gram-positive bacterium *Bacillus subtilis*, gene expression in response to P_i_ concentration is regulated by the PhoPR two-component signal transduction system [35]. In response to limiting availability of exogenous phosphate, *B. subtilis* replaces teichoic acid with a phosphate lacking teichuronic acid polymer to allow scavenging of stored phosphate [25]. Although *Listeria spp.* do not produce teichuronic acids, we similarly hypothesize that the *L. monocytogenes* Δ*phoPR* mutant cannot properly maintain homeostasis of the cell envelope in response to changes in environmental phosphate concentrations, therefore preventing biofilm development (Fig. 3). Correspondingly, using confocal scanning laser microscopy, a significantly thinner biofilm was observed for Δ*phoPR* bacteria compared to the parental 10403S strain (Table 2).

We used our laboratory wild-type strain, *L. monocytogenes* 10403S, as a prototypic *L. monocytogenes* strain for our studies. This strain produces robust biofilms when grown at 35°C in TSBYE medium and subsequently transferred to a minimal nutrient medium and grown in oxygen-depleted conditions. However, it is important to note that other *L. monocytogenes* strains have been shown to produce robust biofilms at different temperatures using alternative growth conditions and are capable of attaching to glass, plastic, and stainless steel [3]. During growth in food processing plants, *Listeria spp*. may encounter rapidly varying temperatures and nutrient availability that can lead to biofilm formation on environmental surfaces and result in potential food contamination. It has been previously shown that *L. monocytogenes* adheres more strongly to polymeric surfaces, as opposed to steel, potentially leading to greater contamination of meat products [12]. Thus, *L. monocytogenes* growth on surfaces can be strongly affected by the type of surface material and the presence of other biofilm-forming microorganisms [12].

Since 2009, the FDA has taken a pro-active surveillance approach to detect and eradicate *L. monocytogenes* from surfaces in food processing plants, therefore reducing the possibility of contamination, since the presence of a small amount of *L. monocytogenes* during food packaging can result in a large inoculum of bacteria being present at the time of consumer consumption [36]. In 2011, the FDA Food Safety Modernization Act was established which enables the FDA to better protect public health by strengthening the food safety system in shifting the focus towards prevention rather than simply responding to food contamination occurrences (http://www.fda.gov/Food/GuidanceRegulation/FSMA/default.htm). Understanding the requirements for surface attachment and biofilm formation by *L. monocytogenes* will facilitate the development of improved mechanisms and standardized procedures for removal of biofilms in food processing environments. Accordingly, the results of these studies are consistent with the aims of the FDA Food Safety Modernization Act in providing support to develop, evaluate, and subsequently implement new methods into the existing FDA environmental sampling inspectional programs to prevent future disease outbreaks from contamination of ready-to-eat foods.

## Supporting Information

S1 File **Table S1, Table S2, and Table S1–S2 References.** Table S1: *Listeria monocytogenes* strains and plasmids used in this study. Table S2: Oligonucleotides used in this study. Table S1–S2 References: References cited in Table S1 and S2.
(PDF)
