# Supplementary material for: Identification of Listeria monocytogenes Determinants Required for Biofilm Formation
Source: PLoS One. 2014 Dec 17;9(12):e113696. doi: 10.1371/journal.pone.0113696 (PMC4269431; doi:10.1371/journal.pone.0113696)
Supplement: S1 File — Table S1, Table S2, and Table S1–S2 References. Table S1: Listeria monocytogenes strains and plasmids used in this study. Table S2: Oligonucleotides used in this study. Table S1–S2 References: References cited in Table S1 and S2. (PDF) [file pone.0113696.s001.pdf]

Table S1. *Listeria monocytogenes* strains and plasmids used in this study.

***Listeria monocytogenes* strain or plasmid**	**Strain genotype or plasmid description**	**Reference**
10403S	Laboratory wild-type strain	[1]
DH-L2052	(DP-L5539) 10403S *Himar1* transposon library	[2]
DH-L2053	10403S *flaA*::*Himar1*	This study
DH-L2054	10403S Δ*dltABCD*	This study
DH-L2055	10403S Δ*phoPR*	This study
pKSV7	Allelic exchange vector for *L. monocytogenes*	[3]
pKSV7 Δ*dltABCD*	Plasmid for in-frame deletion of *dltABCD*	This study
pKSV7 Δ*phoPR*	Plasmid for in-frame deletion of *phoPR*	This study

Table S2. Oligonucleotides used in this study.

**Primer name**	**Sequence (5’-3’)**	**Site[a]/ Reference**
ARB1	GGCCACGCGTCGACTAGTACNNNNNNNNNNGTAAT	[4]
marK3	GCAATGAAACACGCCAAAGTAAAC	[5]
ARB2	GGCCACGCGTCGACTAGTAC	[4]
marK4	CGCCTACGGGGAATTTGTATC	[5]
pKSV7dltABCDfor	AAGTCGACTATCCACATAATGAAGAAATAATCCATGCTCCACAATCCCTGG	*SalI*
pKSV7dltABCDrev	AAGGATCCGCACTCATGCCATCTGCATTTAGTCCATCCTTTGACAG	*BamHI*
dltABCDdelfor	CCATTTTCTGGATGTTCTTTAAAGTAATACATTATTGATATTCCCATTTTTAACAGTCC	
dltABCDdelrev	GGACTGTTAAAAATGGGAATATCAATAATGTATTACTTTAAAGAACATCCAGAAAATGG	
pKSV7phoPRfor	GATCGGATCCGCTATTATTTTGATGTGAG	*BamHI*
pKSV7phoPRrev	GATCGAATTCGTTCATAAGACCAGATTTTCC	*EcoRI*
phoPRdelfor	CATAAAGAAGGGATGGGAAGTAAAAAAGCCTAATAAATATCC	
phoPRdelrev	GGATATTTATTAGGCTTTTTTACTTCCCATCCCTTCTTTATG	

a. The indicated restriction endonuclease site is underlined within the oligonucleotide sequence. N denotes equal concentrations of all four nucleotides.

REFERENCES

1. Bishop DK, Hinrichs DJ (1987) Adoptive transfer of immunity to *Listeria monocytogenes*. The influence of *in vitro* stimulation on lymphocyte subset requirements. J Immunol 139: 2005-2009.
2. Zemansky J, Kline BC, Woodward JJ, Leber JH, Marquis H, et al. (2009) Development of a mariner-based transposon and identification of *Listeria monocytogenes* determinants, including the peptidyl-prolyl isomerase PrsA2, that contribute to its hemolytic phenotype. J Bacteriol 191: 3950-3964.
3. Smith K, Youngman P (1992) Use of a new integrational vector to investigate compartment-specific expression of the *Bacillus subtilis spoIIM* gene. Biochimie 74: 705-711.
4. Garsin DA, Urbach J, Huguet-Tapia JC, Peters JE, Ausubel FM (2004) Construction of an *Enterococcus faecalis* Tn917-mediated-gene-disruption library offers insight into Tn917 insertion patterns. J Bacteriol 186: 7280-7289.
5. Perry KJ, Higgins DE (2013) A differential fluorescence-based genetic screen identifies *Listeria monocytogenes* determinants required for intracellular replication. J Bacteriol 195: 3331-3340.
